# Designing for Dissemination and Sustainability to Promote Equitable Impacts on Health

**DOI:** 10.1146/annurev-publhealth-052220-112457

**Published:** 2022-01-04

**Authors:** Bethany M. Kwan, Ross C. Brownson, Russell E. Glasgow, Elaine H. Morrato, Douglas A. Luke

**Affiliations:** 1 Department of Family Medicine and Adult & Child Consortium for Health Outcomes Research and Delivery Science, University of Colorado School of Medicine, University of Colorado Anschutz Medical Campus, Aurora, Colorado, USA; 2 Prevention Research Center, Brown School, Washington University in St. Louis, St. Louis, Missouri, USA; 3 Department of Surgery (Division of Public Health Sciences) and Alvin J. Siteman Cancer Center, Washington University School of Medicine, St. Louis, Missouri, USA; 4 Parkinson School of Health Sciences and Public Health and Institute for Translational Medicine, Loyola University Chicago, Maywood, Illinois, USA; 5 Center for Public Health Systems Science, Brown School, Washington University in St. Louis, St. Louis, Missouri, USA

**Keywords:** designing for dissemination, designing for sustainability, health equity, impact, knowledge translation, stakeholder engagement

## Abstract

Designing for dissemination and sustainability (D4DS) refers to principles and methods for enhancing the fit between a health program, policy, or practice and the context in which it is intended to be adopted. In this article we first summarize the historical context of D4DS and justify the need to shift traditional health research and dissemination practices. We present a diverse literature according to a D4DS organizing schema and describe a variety of dissemination products, design processes and outcomes, and approaches to messaging, packaging, and distribution. D4DS design processes include stakeholder engagement, participatory codesign, and context and situation analysis, and leverage methods and frameworks from dissemination and implementation science, marketing and business, communications and visual arts, and systems science. Finally, we present eight recommendations to adopt a D4DS paradigm, reflecting shifts in ways of thinking, skills and approaches, and infrastructure and systems for training and evaluation.

## INTRODUCTION

To realize a public health impact of investments in research and evaluation, we should broadly and equitably disseminate and sustain use of evidence-based public health, community, clinical, and health services innovations in diverse settings. However, a return on societal investments in health research is often not seen for a decade or more (5). Passive dissemination of evidence-based interventions is ineffective, resulting in only small changes in the uptake of new practices (12, 91). Barriers to dissemination, sustainability, and impact range from poor fit between health innovations and the context in which they are meant to be used to the research paradigms used to develop and test innovations to cultures and systems that fail to incentivize and support active dissemination and translation of evidence into practice (56, 59, 147, 155; definition from Reference 136). Moreover, greater focus on advancing the science of innovation design and adaptation is warranted to ensure innovations are designed from the outset for diverse reach and feasibility to better address health inequities and improve adoption of health innovations in marginalized and underresourced communities (7).

**Dissemination:**

“An active approach of spreading evidence-based interventions to the target audience via determined channels using planned strategies”

**Sustainability:**

the degree to which an evidence-based program, policy, or intervention can deliver its intended benefits over an extended period of time

**Designing for dissemination:**

the process of ensuring that the products of research are developed to match the...contextual characteristics of the target audience and setting

**Designing for sustainability:**

early planning and design processes designed to increase the likelihood of sustainment of an evidence-based program or practice after initial implementation

Over the past 20 years, the field of dissemination and implementation (D&I) science emerged as part of a collective commitment to accelerate and improve translation of evidence into practice (24). Within D&I science, the concepts of designing for dissemination—and more recently, designing for sustainability—refer to principles and methods for addressing innovation–context fit and the need for early and active dissemination and sustainability planning (17, 71). A key principle in designing for dissemination and sustainability (D4DS) is beginning with the end in mind, that is, to plan for future dissemination, implementation, and sustainability at the outset of a research effort (127). Essentially, D4DS principles and methods aim to enhance the equitable and long-lasting impact of evidence-based innovations on health and well-being. D4DS is now recognized as a key competency for D&I researchers (127).

In this article we suggest that a D4DS perspective can enhance the potential for adoption, sustainability, and ultimately impact of health and health equity. We first summarize the historical context leading to the concepts of D4DS. We then organize and summarize key findings from a narrative literature review according to a schema of dissemination products developed, evaluated, and distributed via various design processes, methods, and frameworks. Finally, we present recommendations and answerable questions for advancing the science of D4DS.

## HISTORICAL CONTEXT

The foundations of D4DS can be traced to diffusion theory, with the earliest concepts originating from the nineteenth-century French judge, Gabriel Tarde, who first proposed the S-curve of diffusion and the importance of opinion leaders (45). In the United States, the seminal event in diffusion was the publication in 1943 of a report on the diffusion of hybrid seed corn in two Iowa communities (142), which contributed to a set of tools and practices for agriculture extension agents (47). The late Everett Rogers formalized many current properties of diffusion in his classic text, *Diffusion of Innovations* (139, 140). Multiple elements from diffusion theory apply to D4DS, including (*a*) the role of specific properties of innovations (e.g., evidence-based interventions) that affect adoption (e.g., costs, advantages over existing practices, compatibility with existing workflow); (*b*) the importance of properties of adopters affecting diffusion, especially the degree of innovativeness within an organization; and (*c*) how social context affects diffusion, including the role of opinion leaders and norms.

There is a well-documented chasm between how researchers disseminate their findings and how practitioners and policy makers learn about and use the latest evidence (26). According to the push-pull-capacity model (1, 43, 54, 124), successful dissemination requires a basis in science and technology (the push), a demand from organizations or the populations being served (the pull), and the delivery ability of public health and health care systems (capacity). Dissemination strategies have often focused too much on the push side of this model while lacking creative approaches and resources to address pull and capacity. The push-pull disconnect between researchers and practitioners was illustrated in the 2002 workshop *Designing for Dissemination* sponsored by the US National Cancer Institute (NCI) (114). A key workshop insight was the endorsement of the importance of active dissemination of the evidence—but neither researchers nor practitioners assumed the responsibility for dissemination activities. Unfortunately, when no one takes leadership and ownership for dissemination or when capacity is lacking, the activity often sinks to low priority in already overstressed systems (27, 79).

A D4DS perspective situates the responsibility for active dissemination within the research enterprise. Guidance on frameworks and necessary systems and processes has emerged, such as with Nutbeam’s (122) 1996 ideas on how to enhance dissemination beyond traditional journal article publications, incentives to reward researchers for translational research, and expanded practitioner training. In 2006, Bauman and colleagues (6) proposed a six-step dissemination framework that highlights the need to (*a*) describe the innovation, (*b*) identify the target audience and the sequence, timing, and format for dissemination, (*c*) define the communication channels, (*d*) determine the role of key policy makers and partnerships, (*e*) identify the barriers and facilitators for dissemination, and (*f*) evaluate the dissemination process.

Although there has been progress in advancing the D4DS perspective, there remains substantial room to improve. A 2012 study of US public health researchers showed only half of respondents (53%) had personnel dedicated to dissemination to nonresearch audiences (27). Only 17% used a model to plan their dissemination activities; 34% always or usually involved stakeholders in the research process. A similar 2018 survey of US and Canadian researchers found some improvement in stakeholder engagement in D4DS processes yet also identified a continuing misalignment between which dissemination methods affect a researcher’s career and which methods affect practice and policy (84).

Theories, methods, and outcomes of evidence-based adoption and implementation have been widely studied (92, 134), whereas relatively less attention has been paid to factors related to successful sustainability of programs and practices postimplementation (154). Programs and practices need to be sustained over time to achieve their desired health impacts and associated outcomes (146). However, many evidence-based programs and policies are not sustained after initial implementation, wasting large amounts of financial, organizational, and social capital (60). Fortunately, new work has started making the case for more systematic study of sustainability, including conceptual development (35, 148), method development (94, 100, 128), and applications to various health disciplines, such as public health, mental health, and health care delivery systems (19). An important audience to consider when designing for sustainability is policy makers—those who make decisions at both local or organizational levels (small p policy) and national or international levels (big P policy) about how and by whom public health and health care should be delivered and financed (23). Designing research products so that they can be effectively communicated to policy audiences helps ensure long-term and sustained impacts of discoveries (9, 69).

## A D4DS ORGANIZING SCHEMA AND LOGIC MODEL

**Figure 1:**
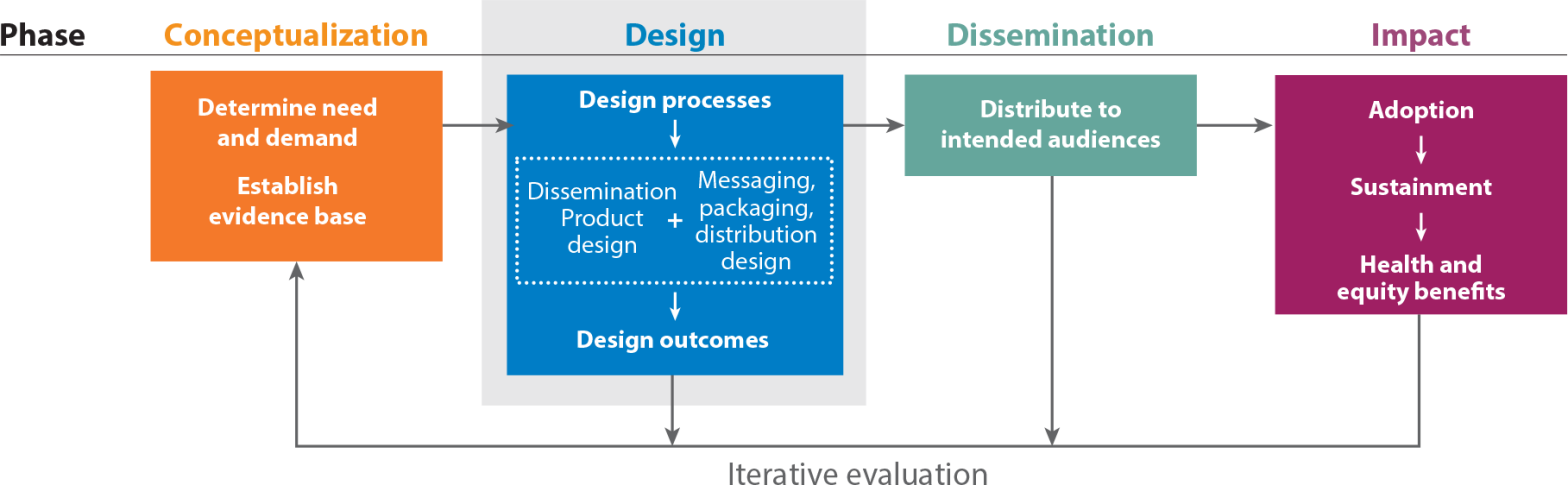
Designing for dissemination and sustainability (D4DS) organizing schema and logic model. A logic model for D4DS includes conceptualization, design, dissemination, and impact phases. Results of a narrative literature review of D4DS are organized with an emphasis on the design phase and corresponding design processes, products, and outcomes.

For this review, we organize the literature and describe a series of exemplar research projects according to a novel D4DS organizing schema (Figure 1). This schema was informed by and expands on previous work to conceptualize processes, products, and system changes needed to support D4DS efforts (27, 126). The schema is represented as a logic model depicting four phases in designing for dissemination in ways that support adoption, sustainment, and equitable impact on health. An initial conceptualization phase determines the need and demand for a solution to a health problem (the pull) and draws on an evidence base of effective strategies for addressing that health problem. To effectively disseminate the evidence in response to the demand, a D4DS approach then considers a design phase (determining the product to be disseminated and how the product will be packaged and delivered) followed by an active dissemination phase (making use of systems and infrastructure—the capacity—to broadly disseminate the product package to intended audiences; the push) and an impact phase (ensuring adoption, sustainment, and ultimate impact on health and health equity). Evaluation occurs at every stage of design and dissemination and is ideally iterative and ongoing to ensure continued fit to context, reach, adoption, sustainment, and equitable impact on health (see the sidebar titled Example D4DS Design Phase Plans).

**Sustainment:**

the degree to which an evidence-based program, policy, or intervention actually remains in use and delivers its intendeds benefits over an extended period of time

A D4DS perspective elevates the importance of the design phase (see the blue box in Figure 1): designing a dissemination product (such as an evidence-based health-related program, device, or service model) and a product messaging, packaging, and distribution plan.

A dissemination product may include one or more of the following:

- evidence (i.e., the generalizable knowledge resulting from the conduct of research and evaluation)
- programs, interventions, and services (i.e., health promotion and/or disease prevention or educational programs, interventions, initiatives, treatments, or services)
- technology and infrastructure (i.e., devices, software, hardware, web-based and other tools and equipment for disease prevention or management, research, evaluation, or educational purposes)
- D&I strategies (i.e., methods, approaches, guides, or materials for dissemination, implementation, and sustainment of effective, equitable, and efficient public health and health care practices in real-world settings)
- policy and guidelines (i.e., local and/or national public health and health care guidelines, practice or implementation standards, and policies emerging from the evidence base)
- methods (i.e., research and evaluation techniques, instruments, tools, models, measures, and/or equipment)

These research products may be packaged in a variety of forms and made available to users through several platforms with messaging tailored to audience needs and perspectives—that is, a messaging, packaging, and distribution plan that also fits the context of intended adoption and use (see the sidebar titled Dissemination Product Examples).

Using various design processes, the dissemination product is designed to fit the context in which it will be adopted and sustained, and the messaging, packaging, and distribution plans fit the information needs and communication channels appropriate for the intended audience. Design processes refer to the methods, frameworks, or approaches used to develop and test the dissemination product and product messaging, packaging, and distribution plans. Design outcomes are those elements relevant for fit to context—such as perceived acceptability, appropriateness, and feasibility or relative advantage, usability, and user satisfaction—and are assessed during the design process.

## A NARRATIVE REVIEW OF THE D4DS LITERATURE

Here we present and organize the literature relevant to the design phase of D4DS: dissemination products, design processes, design outcomes and evaluation, and messaging, packaging, and distribution approaches. The literature described here came from a May 2020 narrative review of published English literature indexed in Ovid MEDLINE, Embase, Web of Science, and Google Scholar. The search included literature referencing dissemination, design, knowledge translation, stakeholder engagement, and/or diffusion of innovation, as well as public health, health science, and clinical research—topics considered likely to identify instances of designing for dissemination in health research. We supplemented papers identified via the designing for dissemination review with additional publications focused on sustainability and sources describing relevant projects identified by the authors.

**Context and situation analysis:**

a formal assessment of the audience, setting, workflows, processes, policies, resources, and systems in which a health innovation is intended to be used

**Social marketing:**

application of marketing principles and techniques to create, communicate, and deliver value in order to influence target audience behavior

### D4DS Design Phase: Design Processes

The design phase of D4DS refers to the active process of developing a dissemination product and planning for its distribution, scale-up, and sustainment, as well as evaluating and iteratively improving design outcomes relevant to ensuring fit to context. We highlight six interrelated major categories of design processes useful for D4DS that emerged from the narrative review: (*a*) participatory codesign and stakeholder involvement, (*b*) application of D&I science theories and frameworks, (*c*) marketing and business approaches, (*d*) context and situation analysis, (*e*) systems, engineering, and complexity science approaches, and (*f*) communication and the arts. These categories are organized by field (marketing, engineering, communication, D&I science) and/or purpose (stakeholder engagement, context analysis).

D4DS efforts may use one or more of these processes. For instance, a D4DS effort may engage stakeholders in a codesign process and conduct a situation analysis within the context of a D&I process framework to design a dissemination product that is then packaged for delivery with graphic design experts and promoted using social marketing (see the sidebar titled Leveraging Multiple D4DS Processes). Furthermore, these processes are not mutually exclusive, nor are they exhaustive of the disciplines and approaches that may be applicable.

#### Design process type 1: participatory codesign and stakeholder involvement.

Ideally, design is done in partnership with the intended audience—such as a participatory, codesign approach—with many arguing that end user or other stakeholder involvement in the design process is essential to equitable adoption and use of health innovations (14, 30, 65, 84). Many types of stakeholders, from multiple cultures and socioecological levels, including members of the public, practitioners, policy makers, and payers, can and should be involved at each stage in the design process (38). Many stakeholder and community engagement methods are available for use in D&I research (107).

The participatory codesign methods category includes techniques such as brokered or deliberative dialogue (129), codesign/coproduction such as experience-based codesign and behavioral design teams (137), group model building and concept mapping (61, 105), consensus approaches such as nominal group technique or Delphi processes (34, 145), and the Double Diamond design approach, which is a four-phase design process popular in the United Kingdom that includes discover, design, develop, and deliver phases (44). Codesign processes benefit from leveraging multisectoral partnerships among academic, industry, health system, and community groups (138).

#### Design process type 2: application of dissemination and implementation science theories and frameworks.

Application of D&I process frameworks can provide structure to the D4DS process. For instance, the IDEAS (Integrate, DEsign, Assess, and Share) framework describes a step-by-step process for designing digital health interventions that is based on design thinking, behavioral theory, user-centered design, and dissemination approaches (112). Developed by the US Department of Veterans Affairs Office of Research and Development Research-to-Real-World Workgroup, the Research Lifecycle framework explicitly incorporates scale-up, spread, and sustain phases of research, depicting the need for a research business plan, common impact metrics, and a sustainability plan as critical steps in the translation of research innovations into common practice (80). Among other D&I frameworks with implementation or planning phases ideal for consideration of D4DS are EPIS (Exploration, Preparation, Implementation, and Sustainment) (111), i-PARIHS (integrated Promoting Action on Research Implementation in Health Services) (90), and the World Health Organization ExpandNet framework for scaling-up (165).

D&I context and determinants frameworks such as diffusion of innovation theory (46) and the PRISM (practical, robust implementation and sustainability model) expansion of RE-AIM (reach, effectiveness, adoption, implementation, maintenance) (55) can guide consideration of multilevel factors that influence dissemination, impact, and sustainability during the design process, informing product features that address barriers and facilitators (17). To enable effective planning for D4DS, Klesges and colleagues (82) illustrated the usefulness of the RE-AIM framework for designing studies with a higher likelihood of future dissemination and uptake. D&I science also contributes methodology and frameworks for planning for adaptation to ensure sustained fit to context, including considerations for cultural adaptations (50, 51). Although still an emerging area, guiding adaptations in a way that maintains the core functions (or principles) of a program, but adapts the form or specifics of how the program is delivered in ways that fit local context, is a promising direction to enhance success (36, 50).

#### Design process type 3: marketing and business approaches.

Marketing strategies also seek to promote adoption and are similarly grounded in diffusion of innovation theory and practice. For example, the field of public health has embraced frameworks and strategies from social marketing (85). Social marketing has shown promise as a dissemination framework for public health efforts (87, 162). Marketing behavior-change techniques can be used to promote broad participation and engagement with dissemination products (52). Best business practices also embrace a multistage development process consistent with D4DS principles: (*a*) problem–solution fit, (*b*) product–market fit, and (*c*) business model fit (15). In the first stage, the developer gathers evidence demonstrating that the innovation is designed to solve an important job to be done, problem, or goal from the adopter’s point of view better than competing alternatives and will generate sufficient value to promote adoption. In the second stage, the developer validates that the innovation does indeed provide that value and that there is a market of potential adopters. In the last stage, the developer ensures the value proposition is embedded in a financially sustainable and scalable business model.

**Systems thinking:**

understanding how things influence one another holistically

The method of iterative customer discovery and value proposition design is foundational in several national training programs designed for the academic research audience: the National Science Foundation/National Institutes of Health I-Corp^™^ training program and its adaptation for clinical and translational scientists [I-Corps@NCATS (National Center for Advancing Translational Sciences)] and the NCI SPRINT (SPeeding Research-tested INTerventions) program supporting sustainability (109, 116). Considering and designing for the consumer perspective—which may include direct marketing to consumers—can serve as a form of collaborative program development (144).

#### Design process type 4: context and situation analysis.

A critical aspect of D4DS is gaining an in-depth understanding of the context and situation in which a product is intended to be used and sustained. Understanding context is foundational to tailoring dissemination products that fit context (8, 118, 132). Context and situation analysis methods yield insights into the unmet needs and perspectives of the intended audience; the existing networks, systems, processes, and workflows into which the product will be integrated; and the resources available to support sustained use (10). Methods such as process mapping, network analysis, needs assessment, ethnography, and discourse analysis fall into this category (53, 95). Qualitative and mixed methods research such as surveys, key informant interviews, and focus groups designed to assess audience needs, circumstances, and perspectives may be used during product design to understand contextual factors thought to influence dissemination, use, and sustainability (143). Of note, context and situation analysis is ideally guided by D&I determinants and outcomes frameworks (as described above). More recent literature on the roles of context stresses the dynamic nature of context and, as noted above, the ability to adapt to changing context as being critical for successful dissemination and sustainability (35, 150).

#### Design process type 5: systems, engineering, and complexity science approaches.

Dissemination and sustainability activities are embedded within complex social, health, organizational, and political systems (97, 121). Systems science approaches such as systems thinking, systems mapping, system dynamics, and human factors engineering have all been used in D4DS endeavors (29, 136, 138, 164). These distinct but related approaches help address the interactive and complex adaptive systems issues in dissemination and sustainment. For instance, systems thinking based on complex adaptive systems with system dynamics mapping has been used to inform large-scale change related to guideline implementation in Canada (13) and various health services outcomes in the US Veterans Administration system (163, 166). A review of system dynamics applications in injury prevention research concluded that building capacity for system dynamics can support stakeholder engagement and policy analysis (115). Other researchers have demonstrated the usefulness of iterative engineering approaches to successful program D&I (135).

Complexity and systems science approaches focus attention on three specific substantive issues: dynamics, heterogeneity, and interactivity. First, the organizations and communities that are adopting and implementing new evidence-based practices are dynamic, not static. Systems perspectives can help focus attention on these dynamics, including feedback loops, indirect effects, and unintended consequences, as well as the need for program adaptation over time (35). Second and third, these complex systems are composed of heterogeneous actors (e.g., patients, health care providers, regulatory agencies, commercial businesses) that interact with one another. Systems tools that reveal and explore these interactions, such as social network analysis and systems mapping, are thus useful for the dissemination design process (98, 158). Systems thinking can inform dynamic and complicated processes that ensue once a scientific discovery moves into the real world. For example, policy resistance is the general tendency for policy interventions to be weakened or defeated by the system’s response to the intervention itself (58, 153). A vivid recent example of this has been the pushback by communities and government leaders on the implementation of evidence-based vaccination and masking policies to combat the coronavirus disease 2019 (COVID-19) pandemic (149). Historically, the tobacco industry and other commercial and political allies have used tools such as grandfathering, preemption of local policy implementation, and delaying full policy implementation to weaken the effects of evidence-based tobacco control policies (99). By preparing for policy resistance in the design process, dissemination products can lead to greater adoption and sustainability and subsequent health impacts.

**Arts-based knowledge translation:**

using various art genres, such as visual arts, performing arts, creative writing, and multimedia including video and photography, to communicate research

#### Design process type 6: communication and the arts.

Methods from the fields of communication, media production, advertising, journalism, and graphic design are especially relevant to the design of dissemination product messaging, packaging, and distribution plans (18, 104). Arts-based knowledge translation (3, 88) has been proposed as a viable strategy for dissemination to health care provider and consumer groups. Use of visual graphics can support communication with and translation of complex science concepts to target audiences (73, 119). For instance, this video by the University of Colorado Record Linkage team (https://youtu.be/kt9u5omGwsY) uses graphic storytelling to explain research data privacy and security concepts. The *SAGE Handbook of Visual Research Methods* (102, 130) describes a plethora of visual research techniques for public engagement and communication, including visual media production, photovoice, visual ethnography, anthropological filmmaking, multimodal strategies, and making arguments with images, many of which have great potential for use in D4DS efforts.

### D4DS Design Phase: Messaging, Packaging, and Distribution of Dissemination Products

As shown in Figure 1, the way in which a dissemination product is messaged, packaged, and distributed to intended audiences is an aspect of the D4DS approach parallel to, but distinct from, the design of the dissemination product itself. Messaging, packaging, and distribution plans should be aligned with how the intended audience best receives information and should leverage existing and familiar distribution channels, platforms, and systems of communication and influence (25). Presenting data in ways that are engaging and easily understood is a hallmark of effective evidence communication to many audiences. For instance, data visualization techniques and tools have been used to communicate complex health science data in areas such as cancer genome data (83) and HIV population dynamics (16) (see the sidebar titled Packaging and Distribution of a Dissemination Product).

Marketing and communication methods are particularly relevant to message design. The customer discovery and value proposition design approach, a method taught in the I-Corps@NCATS training program (117), yields validated message framing about the value of a dissemination product on metrics most important for the target audience and in the context of competing alternatives (125). Customer discovery and value proposition design has been used to develop positioning and key claims (e.g., business pitches) and distribution plans for dissemination products such as behavioral interventions (78, 123) and research networks (109, 110).

End user preferences for how evidence should be packaged and delivered need to be considered, as preferences can vary by audience (151, 156). For instance, Crick & Hartling (41) described an evaluation of stakeholder preferences for sharing evidence from systematic reviews, showing that patients, caregivers, and nurses preferred infographic-style reports while physicians preferred critical appraisal–style reports. For policy audiences, research should be presented in brief formats with talking points, reflecting needs relevant to current policy priorities, and tailored to meeting policy needs based on local data (4).

Packaging dissemination products have taken multiple forms, such as web-based knowledge translation platforms (11, 42), evidence search and synthesis tools (48, 68), and professional learning and training platforms (64). Implementation guidance products include implementation guides (39, 152), knowledge management tools for informing evidence-based decision making (159), and multimedia informational briefs and education materials (67). Some particularly creative examples of public dissemination strategies include the use of applied theater to share findings from a study of prenatal genetic screening (74) and an arts-based immersive concept exhibit to disseminate evidence from more than 500 scientific studies of menopausal hot flashes (32, 33).

As would be expected in the digital age, packaging and distribution often leverage computer and information technology, such as the internet. Twenty years ago, the US NCI funded four Digital Divide Pilot Projects, which were intended to be models for larger-scale efforts, to test the use of computer technology and the World Wide Web for the dissemination of cancer education to the public (86). In the time since, interest has grown in the use of the internet and social media for research dissemination and health communication with the public and clinical and public health professional audiences (37, 57, 75). Although it can be challenging to address health misinformation and disinformation on social media and other forms of mass media (62), the ability to disseminate to the public at such a large scale has been unprecedented. A 2020 review of social media use for knowledge translation and education of physicians and trainees identified over 450 examples of the use of platforms such as Twitter, Facebook, blogs, and podcasts for medical education (37). An increasingly popular web-based approach to health sciences knowledge translation is collaborative writing applications (CWAs), such as wikis or learning management systems (2). A 2013 scoping review on the use of CWAs for knowledge translation showed that CWAs are often used by members of the public and researchers for health information seeking and by health professionals for peer-to-peer communication (2).

**Collaborative writing application (CWA):**

social media software platform that allows multiple users to create and edit content

### D4DS Design Phase: Design Outcomes and Iterative Evaluation

To ensure a dissemination product exhibits fit to context, the design process should include evaluation of design outcomes such as perceived acceptability, appropriateness, and feasibility (161); implementability (76); and sustainability at the setting level (77) and usability, usefulness, and user satisfaction at the user level (133). Other design outcomes may include needed adaptations to fit changes in context over time or when translating a product for use in a new setting (35). Evaluation and research methods for assessing design outcomes range from user testing (e.g., system usability, user satisfaction, user engagement) (141) to randomized trials and other research designs appropriate for testing D&I strategies (103) and can use quantitative and/or qualitative methods. Ideally, researchers rapidly and iteratively assess design outcomes on the basis of successive prototypes or minimum viable products before moving on to large-scale testing. For instance, the Multiphase Optimization STrategy (MOST) approach (63)—often used to guide design and testing of multicomponent behavioral interventions—includes an optimization phase that can benefit from rapid and iterative testing of early prototypes (see the sidebar titled Iterative Design and Evaluation).

Upon evaluating design outcomes, it may become clear that the envisioned dissemination product does not fit the context or target audience needs, necessitating a pivot or possible abandonment of the idea altogether. This is a valuable outcome, as it can prevent continued investment in a product unlikely to be broadly adopted. Public health officials, clinicians, and health care organizations often waste time and resources on adapting and adopting solutions that are ultimately not scalable nor financially sustainable, thereby providing a negative feedback loop and reducing motivation to engage in the implementation of future solutions (72) (see the sidebar titled Example Pivots in the D4DS Process).

### D4DS Dissemination Phase: Capitalizing on Systems and Infrastructure for Dissemination

To accelerate D4DS, systems and infrastructure need to be in place (27). Applying the push-pull-capacity model described above, push issues include providing incentives and infrastructure for researchers to actively disseminate to nonresearch audiences (e.g., a dedicated communication/dissemination unit, promotion/tenure guidelines) (108, 155), hiring faculty with practice and policy experience (155), making dissemination to nonresearch audiences a scorable part of the grant review process, and including practice and policy stakeholders in the grant review process (93). To enhance the pull for research among practitioners, organizations must focus on changing their climate and culture. For example, evidence-based organizations in public health provide ready access to research-tested products, leadership applies principles of evidence-based decision making, and innovation is encouraged in day-to-day activities (22).

Capacity for D4DS can be supported in several ways. For example, infrastructure that facilitates bidirectional communication between community practice and research to support evidence dissemination includes practice-based research networks (70). Personnel such as knowledge brokers and implementation facilitators support health settings by integrating new knowledge of practices into daily work (49). Health sciences libraries can support dissemination activities such as data visualization and dissemination product archiving and cataloging (66).

### D4DS Impact Phase: Assessing Adoption, Sustainment, and Equitable Impact

As our D4DS schema suggests (Figure 1), longer-term health and equity impacts are the natural goal of effective design of dissemination, implementation, and sustainability products and processes. Conversely, a lack of attention to the specific aspects of design, as described above, impedes our ability to move from evidence to impact and exacerbates health disparities. Underlying a D4DS approach is a need for consideration of equity concepts, methods, and activities in all phases of research. Health equity is a critical part of the goals of positive health impact (see the right side of Figure 1). That is, part of the conceptualization of D4DS, as ensuring fit to context means ensuring dissemination products are culturally appropriate, can be used in low-resource settings, can align with the strengths and assets of the intended audience and setting, and can affect outcomes that matter to local stakeholders (120, 150). Although recognition of health disparities has a long history, we are only now formally integrating concepts of social justice, health equity, and social determinants of health into D&I theories, methods, and practices (20, 28). Through authentic engagement of stakeholders in design, implementation, and evaluation of health innovations and use of science communication approaches sensitized to a diversity, equity, and inclusion lens, dissemination products are more likely to reach historically marginalized and underserved communities (28, 31).

An unfortunate aspect of traditional graduate training in public health, mental health, and clinical science research programs is the overemphasis on immediate scientific outputs (i.e., papers, conference presentations, grants) relative to training for how to frame, communicate, and assess longer-term health and social benefits of health research. Government funding agencies and university systems in many parts of the world, particularly in the United Kingdom and Commonwealth countries (e.g., Research Impact Canada, the United Kingdom’s Research Excellence Framework, University College Dublin’s framework for research impact assessment), have formally started to adopt policies that support (and even require) more attention to research impact. New impact frameworks and tools focus specifically on health and clinical sciences research (89, 96) and health equity (106). For example, the Translational Sciences Benefits Model (TSBM) suggests that the long-term benefits of health and clinical sciences research will be seen in predominantly four areas: clinical improvements, community and population health improvements, new economic activities and benefits, and enhanced policies and laws (96). These frameworks provide some conceptual structure and goals for the early design phases in health research. That is, just as we can design for dissemination and sustainability, we can also design for impact.

## RECOMMENDATIONS TO ADVANCE THE FIELD OF D4DS

In Table 1, we articulate eight D4DS recommendations to advance the field and address the need for improved adoption, sustainment, and equitable impact of health innovations. For each recommendation, we propose specific actions and answerable questions to be tested in comparative effectiveness research.

## CONCLUSION

Most health research is neither translated into practice nor sustained owing to poor innovation–context fit as well as a lack of resources to support it and a lack of emphasis on active dissemination. A D4DS perspective places the responsibility for active dissemination in the scope of work of the research enterprise and related partners. To support the D4DS perspective, we offer an organizing schema that emphasizes the design phase of developing a dissemination product and a messaging, packaging, and distribution plan. We specify design processes that can be used individually or in combination during the design phase. Yet to advance the science and practice of D4DS, we should reorient toward a mindset of beginning with the end in mind and consider from the outset the needs and demands of the intended audience and setting for use of research innovations. Finally, those seeking to implement our recommendations should advocate for changes to promotion and tenure criteria that emphasize dissemination, sustainment, and impact beyond academic journal articles.

## Figures and Tables

**Table 1:** D4DS: recommendations and answerable questions

Recommendation	Explanation	Specific action or answerable question
**Shifting ways of thinking: How to view the world from a D4DS perspective**		
Recommendation 1: Begin with dissemination, sustainment, and equitable impact in mind	It is not enough to begin with anticipated health outcomes in mind—begin by asking, Who will influence the decision to adopt and sustain? How will this work ensure equitable impact?	To what extent do specific activities designed to enhance dissemination, sustainability, and equity yield improved health impacts?
Recommendation 2: Prioritize the needs and perspectives of diverse stakeholders at every stage of the process	Involving stakeholders from multiple perspectives, including potential adopters, will help anticipate challenges; keeping stakeholders involved throughout the process should improve quality of adaptations.	To what extent does ongoing involvement—in different ways and at multiple points in time—produce greater impact than more modest or one-time stakeholder engagement?
Recommendation 3: Appreciate the value of a rapid and iterative approach and the need for periodic adaptation	Anticipate and plan for the need to adapt programs or strategies in response to dynamic context over time.	In what ways do approaches that specifically include multiple assessment points for review of results to date and iterative adaptations yield enhanced impact?
**Shifting skills and approaches: What we need to do differently to realize the promise of D4DS**		
Recommendation 4: Incorporate team science and systems science principles and practices	D4DS is a collaborative enterprise and produces products that influence systems of care and health. Team and systems science best practices can help ensure that teams work well together and that they can produce better products.	To what extent do programs and products that incorporate team science and systems science methods produce greater impact?
Recommendation 5: Employ health communication techniques tailored to the intended audience	One size does not fit all, and framing how programs and products are discussed and promoted has a big impact on adoption.	Do products distributed to intended audiences using health communication and audience-targeted strategies produce greater adoption?
Recommendation 6: Evaluate adoption, equity, and sustainment at scale (21)	Transparent reporting and rigorous evaluation of adoption, equity, and sustainment impacts and relationships among them using both randomized and nonrandomized designs are needed (21).	To what extent can the field be advanced by investigations that provide full reporting on all three of these impacts rather than on health impacts only?
**Shifting training and evaluation systems and infrastructure: What we need to build to support shifting views, skills, and approaches**		
Recommendation 7: Establish and promote training programs that acculturate trainees to the D4DS perspective and teach D4DS skills	Training in key issues described in this article (e.g., communications training, systems science, user-centered design, in-depth training in stakeholder engagement) helps promote equity.	To what extent do training programs and activities that include key D4DS competencies produce better, more sustainable results than those that do not?
Recommendation 8: Provide resources to assist programs and policies that inform D4DS and develop practice-based evidence (28)	The above recommendations require support and funding. Infrastructure is needed to accommodate emerging D4DS lessons learned.	To what extent do programs and trainings that provide targeted resources and specific responsibilities for D4DS and continuous evaluation produce more sustainable and equitable impacts?

Abbreviation: D4DS, designing for dissemination and sustainability.

## EXAMPLE D4DS DESIGN PHASE PLANS

Developed and tested using a multiphase approach, the Program Sustainability Action Training Program is a training program designed to improve the sustainability of evidence-based state tobacco control programs (160). The experiential learning framework guides development of a training curriculum protocol and measures to assess long-term program sustainability. Following a group-randomized trial to test the effectiveness of the training and technical assistance in improving sustainability outcomes in 24 state tobacco control programs, final training curricula materials are actively disseminated to a broader public health audience.

Ending the HIV Epidemic in the U.S. (EHE) is a large-scale plan by the US Department of Health and Human Services to address implementation gaps in the use of evidence-based interventions for preventing and treating HIV (113). EHE includes a well-designed dissemination system for distributing metrics on population health outcomes, implementation, and disparities to key stakeholders. Expected outcomes include reduced health disparities, creation of actionable information, and rapid reach/timeliness of population health metrics via publicly available dashboards with interactive data visualizations and social media integration.

## DISSEMINATION PRODUCT EXAMPLES

An example of a policy dissemination product, KTPMalawi is a knowledge translation platform consisting of communities of practice that codesign evidence briefs to guide health policy in Malawi (11). Developed using a rapid stakeholder mapping exercise, capacity building workshops, engagement of policy makers in structured deliberative dialogues, and collaboration with public media, KTPMalawi has been applied to evidence-based policy making efforts related to chronic disease management and drug supply chains. An example technology dissemination product, the F-Words Knowledge Hub is a web-based resource for families and service providers to inform people about the F-words in childhood disability (function, family, fitness, fun, friends, future) (42).

## LEVERAGING MULTIPLE D4DS PROCESSES

The Health TAPESTRY (Teams Advancing Patient Experience: Strengthening Quality) intervention consists of patient home visits and e-health technology to support interprofessional care for older adults with multiple chronic diseases in Ontario, Canada. The intervention was developed using a modified version of a persona-scenario participatory codesign method (157). An analysis of the intended context used a sequential mixed methods design, with a sustainability questionnaire followed by qualitative interviews, to assess potential for sustainability and scalability, reproducibility, feasibility, and perceived value (77). Questionnaire results showed the intervention was potentially sustainable with improvements to staff involvement and training plus sustainability infrastructure; interviews revealed several themes regarding recommendations for promoting intervention sustainability and scalability, noting that other jurisdictions beyond the pilot site were not ready to adopt Health TAPESTRY. A planned randomized control trial to test reproducibility, implementation, and value for money is under way as of 2020, guided by the D&I frameworks RE-AIM (reach, effectiveness, adoption, implementation, maintenance) and normalization process theory (101).

## PACKAGING AND DISTRIBUTION OF A DISSEMINATION PRODUCT

The MOVE! Weight Management Program for veterans in the US Veterans Health Administration (VHA) was designed to translate best practice guidance and evidence on weight loss into practice (81). The program was packaged in the form of a toolkit consisting of patient handouts, promotional brochures, clinical references, and administrative manuals (https://www.move.va.gov/ReferenceTools.asp); marketing materials (e.g., posters, banners, pens); and online discipline-specific training modules about weight management with continuing education credit. All VHA networks and medical centers received the packaged intervention. Established VHA policy and clinical practice guidelines now require weight management programs, with MOVE! recommended.

The iCHAMPSS (interactive CHoosing And Maintaining Effective Programs for Sex Education in Schools) program is an online decision support system to help school districts adopt, implement, and maintain sexual health evidence-based practices (131). Guided by the program design and program production steps of the intervention mapping approach, and with support from information technology developers, graphic designers, and video producers, the iCHAMPSS team planned, drafted, pretested, refined, and produced program materials. Materials include a website with a video-based tutorial, a staging tool, a tool library, and an online discussion board.

## ITERATIVE DESIGN AND EVALUATION

Deciding on Treatments Together (DOTT) is a decision aid for stroke survivors developed using a user-centered, data-driven design approach including multistakeholder focus groups and interviews to identify needs and priorities, collaborative design, and iterative prototyping (133). Usability and acceptability evaluation (surveys and interviews) of the DOTT prototype showed high usability scores among both stroke survivors and general practitioners; although there was perceived relative advantage compared to usual strategies for treatment decision making, feasibility and appropriateness concerns included time burden and limited scope of patient needs considered.

## EXAMPLE PIVOTS IN THE D4DS PROCESS

Craven et al. (40) set out to create a visualization toolbox that could be applied via digital platforms (web- or app-based) to support adults with lived experience of mental health difficulties present and track their personal well-being in a multimedia format. Yet by applying the Double Diamond design participant codesign methodology, the researchers determined that this approach would not be appropriate. Assessment of personal data visualizations created by participants in a codesign workshop revealed inconsistency with visualizations currently described in the literature and those available in existing mental health apps; this finding suggested an authoring approach to visualizations, in which apps allow users to generate their own personalized content, would be preferred by users. The team pivoted to explore delivery of the insights with “experts-by-experience” by leveraging existing tools for user-generated visualizations (e.g., Bitmoji, Mental Snapp, Snapchat) as part of training courses on use of digital tools for mental health recovery.

## SUMMARY POINTS

1. The historical context of designing for dissemination and sustainability (D4DS) is based in part on diffusion theory and a push-pull-capacity model.
2. We present a novel D4DS organizing schema that situates a design phase within a logic model format, depicting a path from conceptualization to design to dissemination to impact. The design phase considers both a dissemination product [such as evidence, programs and interventions, technologies, policies and guidelines, dissemination and implementation (D&I) strategies, and methods] and messaging, packaging, and distribution plans.
3. A multipronged approach to design integrates processes from multiple disciplines (e.g., D&I science, systems science, business and marketing, communications and the arts), formally and iteratively assesses and aligns with the context of intended use, and engages stakeholders from the intended audience and setting in all aspects of planning, implementation, and dissemination.
4. The D4DS design phase fits within multiple D&I science and design frameworks that include planning, preparation, preimplementation, and iterative testing and adaptation phases.
5. A D4DS approach should begin with the end in mind, specifically in terms of what is needed to promote high reach, to advance health equity, to achieve a broad set of translational research impacts, and to be sustainable.
6. Recommendations to advance the field of D4DS include the need to shift our thinking to prioritize the needs of stakeholders and value a rapid, iterative approach that welcomes adaptations; to shift the skills and approaches we use in research to emphasize team and systems science; and to allocate resources for building infrastructure and systems that prepare the next generation to approach research from a D4DS perspective.
